# Fear of Recurrence in Chinese Cancer Patients: Prevalence, Correlates, and Network Analysis

**DOI:** 10.3389/fpsyt.2022.803543

**Published:** 2022-02-07

**Authors:** Xian Luo, Wengao Li, Yu Chen, Hengwen Sun, Gerry Humphris, Ting Liu, Jingying Zhang, Yuan Yang, Bin Zhang

**Affiliations:** ^1^Department of Psychiatry, Southern Medical University Nanfang Hospital, Guangzhou, China; ^2^Department of Psychiatry, 999 Brain Hospital, Guangzhou, China; ^3^School of Nursing, Southern Medical University, Guangzhou, China; ^4^Department of Radiotherapy, Cancer Center, Guangdong Provincial People's Hospital (Guangdong Academy of Medical Sciences), Guangzhou, China; ^5^Department of Health Psychology, School of Medicine, University of St Andrews, St Andrews, United Kingdom; ^6^Guangdong Mental Health Center, Guangdong Academy of Medical Sciences, Guangdong Provincial People's Hospital, Guangzhou, China

**Keywords:** Chinese, fear, network analysis, psycho-oncology, recurrence, cancer

## Abstract

**Background:**

Fear of cancer recurrence (FCR) is a significant issue for most cancer patients. Until now, a detailed investigation of the structure of FCR and the interaction among its constituent elements is lacking. This study aims to investigate the phenomenon of FCR by means of network analysis in Chinese cancer patients.

**Methods:**

This is a multi-center, cross-sectional study that included 996 cancer patients from southern China. All participants were assessed by the 7-item Chinese version Fear of Cancer Recurrence Scale (FCR-7). Multivariate logistic regression, and network analyses were conducted. Central symptoms (nodes) in the FCR network were identified.

**Results:**

Among the 996 patients, 543 (54.52%) reported moderate FCR, and 137 (13.76%) reported high FCR. Chemotherapy (OR = 2.954, *P* = 0.016), and childhood severe illness experience (OR = 2.331, *P* = 0.016) were positively associated with high FCR, while higher monthly income (OR = 0.403, *P* = 0.046) was negative associated with high FCR. The node #FCR2 (*Worried/anxious about recurrence*) was the most central node within the FCR network (Strength = 1.190), while node #FCR6 (*Examining for physical signs*) was the least central node (Strength = 0.373). The edge FCR1-FCR2 (“*Afraid”-“Worried/anxious”*) was the thickest and most saturated edge in the network. After controlling for age and gender, an almost identical network was obtained with respect to edges magnitude and strength.

**Conclusion:**

Fear of recurrence is a frequently reported issue among Chinese cancer patients. Patients with chemotherapy and childhood severe illness experience were more vulnerable and should be particularly monitored. Compared to behavioral component (i.e., body checking, overscreening and overtreatment) and cognitive component (i.e., intrusions), emotional component (i.e., worry/anxious) is more central to identify FCR and might be potential targets for further interventions.

## Introduction

Fear of cancer recurrence (FCR) is a significant issue for most cancer patients, and it is a key unmet need among this population (1, 2). As one of the most prominent and common existential difficulties facing cancer patients, FCR could lead to negative physical (i.e., pain, fatigue, and insomnia), psychological (i.e., depression, and anxiety) health outcomes and might last even for years after active treatment (3–5). There are four features that have been identified as core characteristics of clinical FCR: (1) high levels of preoccupation; (2) high levels of worry; (3) persistence; and (4) hypervigilance to bodily symptoms (6). However, until now, there is no consensus upon “gold-standard” diagnostic criteria, assessment, or clinical cutoff of FCR. Therefore, valid estimates of the rate of FCR remain inconsistent (7). In 2013, Simard's review reported that about 22 to 87% of the cancer survivors report moderate to high levels of FCR, while around 15% of them report high or clinical levels of FCR across different cancers (1). A 10-year longitudinal study showed that though FCR tended to decline slightly over time, yet 17% of cancer survivors continue to report high FCR (8).

Recent studies have consistently found that younger age (9), female gender (10), mastectomy (11), radiotherapy (12) and chemotherapy (13) were significant predictors of higher level of FCR (14). In order to manage FCR, cancer patients may develop maladaptive behaviors, such as overscreening or overtreatment (i.e., repeated medical tests), reassurance seeking (i.e., unscheduled visit with their doctors), and avoidance behaviors (i.e., social isolation, substance use, or refusing follow-up clinical visits) to assert control over the uncertainty of their health (4, 5). Several evidence-based interventions have been developed to help those individuals who suffer from severe FCR, including cognitive behavioral therapy (CBT), meditation, relaxation, and creative arts, and have showed small-to-medium effect sizes (5, 15).

FCR is a complex phenomenon among cancer patients with significant clinical consequences, however, little is known about the inner structure of FCR. Previous studies usually focused on FCR total score singularly. However, examining the associations among individual FCR symptoms/items may provide new insights for us. Network analysis estimates the unique associations between each pair of measured symptoms while controlling for all other symptoms and shrinking potentially spurious associations to zero (16). Analyzing FCR symptoms from the perspective of network analysis allows better understanding of which symptoms might be particularly central to the experience of FCR, and which associations (edges) between symptoms (nodes) function as the main pathways of the network (17). It could be highly valuable for better describe psychological constructs and to develop hypotheses for future study (18).

As an integrative approach to study the structure of psychopathology, in recent years, network analysis has been increasingly used in the field of psychology and psychiatry among different populations. For example, Mullarkey et al., used network analysis to study adolescent depression using the Children's Depression Inventory, and found that “self-hatred”, “loneliness”, “sadness”, and “pessimism” were the most central symptoms among adolescents (19), while Murri et al., identified “death wishes” as the “backbone” that sustains depression in late-life, using the EURO-D scale (20). Besides depressive disorders, clinical researchers also applied network analysis to better understand the structure and central symptoms of anxiety disorders, hopelessness, Obsessive compulsive disorder, and eating disorders (21–23). In network analysis, higher centrality index values are representative of greater importance. A previous study found that, compared to patients who endorse more peripheral symptoms of depression, those who endorse more central symptoms of depression at baseline are at increased risk of experiencing major depressive disorder in the following 6 years (24).

FCR has received growing attention in research with many cancer survivors of various cancer diagnoses. However, a detailed investigation of the structure of FCR and the interaction among its constituent elements is still lacking. Therefore, the main aim of the current study is to investigate the phenomenon of FCR by network analysis among Chinese cancer patients. Meanwhile, the prevalence and associates of FCR will be examined. To do so, we aim to closely study the 7-item Fear of Cancer Recurrence scale (FCR7), which is a well-developed and widely-used tool for FCR in different cancer populations (25). In the current study, a network model on the FCR7 items will be generated and the centrality index values will be calculated.

## Methods

### Participants

Study participants were newly diagnosed cancer patients who were seen consecutively at the cancer center in Southern Medical University Nanfang Hospital, Guangdong Provincial People's Hospital, and Guangzhou Women and Children's Medical Center. Participants were recruited from September 2018 to September 2019. Inclusion criteria were: (1) age ≥ 18 years, (2) have a diagnosis of cancer within 6 months, (3) be able to read and write Mandarin or Cantonese; (4) provide written informed consent. Participants were excluded if they had severe physical and/or cognitive impairment. This study was performed in accordance with the Helsinki standard and approved by the Hospital Ethics Committee (Ref No: NFEC-2018-038). Detailed study procedure has been introduced elsewhere (26).

### Measurements

#### Demographic and Clinical Characteristics

Basic demographic and clinical information were gathered using a specially-designed case record form (CRF), such as current age, gender, marital status, education, personal income level, type of cancer, primary treatment (surgery, chemotherapy, and radiation treatment), family cancer history, medical comorbidities, childhood adversity experience (i.e., sexual abuse, bullying, traffic accident, or natural calamities) and childhood severe illness experience (i.e., childhood cancer, or traumatic injury).

#### Fear of Cancer Recurrence

The 7-item FCR questionnaire is used to assess cancer patient's recurrence fears and has been used with patients with breast, colorectal and head and neck cancer in a variety of clinical centers in the UK (27). The reliability of the questionnaire is good with an internal consistency of 0.92 (25). The statistical 60th (score = 17) and 90th (score = 27) percentiles have been regarded as levels for “moderate” and “high” reports of patient's FCR respectively. The Chinese FCR-7 showed good psychometrics properties (Cronbach's alpha = 0.87) (28).

### Statistical Analyses

#### Multivariate Logistic Regression Analysis

Statistical analyses were performed using SPSS version 24.0 (Armonk, MY, USA). To characterize all study variables, descriptive statistics were first calculated. Then, multivariate logistic regression analysis with “enter” method was used to identify potential variables associated with high FCR. High FCR (total score >27) was the dependent variable, and all tested variables were entered as independent variables. Alpha was set at 0.05 (two-tailed).

### Network Estimation

#### Network Structures

Network analyses were conducted with the R program using the “*bootnet”* (16) and “*qgraph”* (29) packages. In network analyses, symptoms/items are identified as “nodes”, and correlations between individual symptoms are defined as “edges” (21, 30). The thickness of the edge indicates the strength of the association between nodes. The color of the edge indicates the direction of the association (e.g., a green line indicates a positive association, while a red line indicates a negative one). Nodes with stronger and more frequent associations with another node are placed closer to each other and more concentrated in the network.

The network structure of FCR was estimated using the Enhanced Least Absolute Shrinkage and Selection Operator (eLASSO) method (31). Furthermore, to determine the optimal network model, the extended Bayesian information criterion (EBIC, a widely applied approach in model selection) was utilized and 0.5 was set as the default tuning parameter (32). In the current model, we used the “*estimateNetwork*” function (16) to assess the network model, and “*EBICglasso*” was set as the default method. In summary, by choosing “*EBICglasso*” as the default method and setting 0.5 as the default tuning parameter, we achieved two goals: (1) the links between two symptoms were not spurious because of other nodes (i.e., conditional independence association), and (2) small link/edge coefficients were reduced to zero, hence allowing the network model to be interpreted with relative ease.

#### Central Symptoms

To highlight which symptoms may be most influential in the FCR network, *Strength, Betweenness* and *Closeness* indices were calculated. *Strength* is the sum of the weight of all direct connections between a specific node and others, indicating the importance of an individual variable. *Betweenness* indicates the number of shortest paths connecting any two symptoms, reflecting the importance of the connection of a variable to other variables, while *Closeness* is computed as the inverse of the sum of the total length of all the shortest paths between a specific node and the rest of the network; therefore, it reflects the strength of the indirect connections of a variable to other variables. Centrality plots were created to represent these indices. For each index, high values reflect great centrality in the network. In the current study, centrality indicators were calculated with the “*centralityPlot*” function, and they are reported as standardized values (z scores).

#### Network Stability and Accuracy

We tested the stability and accuracy of our network via both case-dropping bootstrap test for centrality stability and bootstrap confidence intervals (CIs) for the accuracy of network edges. First, we applied a case-dropping subset bootstrap to compute the correlation stability (CS) coefficient (1,000 permutations). It has been suggested that the correlation coefficients between the original indices and indices based on a case-subset network should be at least 0.7 (default) or higher (with 95% probability) (16). A CS coefficient (correlation = 0.7) represents the maximum percentage of sample cases that can be dropped from the original full cases to retain a correlation of 0.7 in at least 95% of the samples. Generally, the CS coefficient is required to be above 0.25, indicating that 25% of the sample can be dropped while maintaining similar centrality indices (16). Second, we performed nonparametric bootstrapping to estimate the 95% CIs of the edge values to evaluate the accuracy of the edge weights. Larger CIs indicate reduced precision in the estimation of the edges, whereas narrower CIs imply a more trustworthy network (19). Furthermore, in this network analysis, we also used a bootstrap differential test to evaluate the differences in network properties (i.e., differences between the edge weights and the node strengths) (19).

#### Network Model Controlling for Age and Gender

Previous evidence indicated that age and gender might influence FCR. Younger age and female gender are significant risk factors of high FCR (1, 9, 10). Therefore, in line with previous similar studies (23, 33), the network model and the local structure indexes were re-estimated, after controlling for age and gender in this study.

## Results

### Participants and Prevalence of FCR

In total 1,219 cancer patients were invited to participate in the study with 996 providing consent and completing all assessments. Comparison of patients with complete data (*n* = 996) vs. the rest of the invited sample (*n* = 223) revealed no significant differences in gender and age. Demographic and clinical characteristics of included participants are reported in Table 1. The mean age of the final sample was 48.04 years (SD = 11.71). In all, 543 cancer patients (54.52%, 95% CI: 51.42–57.62%) reported moderate-level FCR, and 137 (13.76%, 95% CI: 11.61–15.90%) suffered from high-level FCR. The overall mean and standard deviation (SD) of the FCR sum score is 19.93 (SD = 6.35) (Supplementary Table 1).

**Table 1:** Multivariate logistic regression of variables associated with high FCR.

**Variable**	**Category**	**Total (%)**	**OR**	**95%CI lower**	**95%CI upper**	***P***
Age	≤ 39 years	246 (24.7)	Ref	-	-	-
	40–60 years	556 (55.8)	0.666	0.404	1.098	0.111
	≥ 60 years	194 (19.5)	1.328	0.727	2.428	0.357
Gender	Male	98 (9.8)	1.061	0.473	2.380	0.887
Cancer Type	Breast	803 (80.6)	Ref	-	-	-
	Lung	109 (10.9)	1.646	0.755	3.586	0.210
	Colorectal	84 (8.4)	1.325	0.610	2.878	0.476
Marital status	Single	72 (7.2)	Ref	-	-	-
	Married	852 (85.5)	0.623	0.274	1.418	0.260
	Divorced	39 (3.9)	0.596	0.157	2.258	0.446
	Widowed	33 (3.3)	0.697	0.195	2.489	0.579
Education level	High school or below	655 (65.8)	Ref	-	-	-
	Undergraduate	261 (26.2)	1.278	0.734	2.224	0.385
	Postgraduate or above	80 (8.0)	1.767	0.890	3.511	0.104
Personal monthly income	<3,000 RMB	461 (46.3)	Ref	-	-	-
	3,000–5,000 RMB	258 (25.9)	0.390	0.233	0.652	**<0.001**
	5,001–10,000 RMB	196 (19.7)	0.432	0.229	0.812	**0.009**
	> 10,000 RMB	81 (8.1)	0.403	0.165	0.983	**0.046**
Surgery		908 (91.2)	1.420	0.683	2.953	0.347
Chemotherapy		880 (88.4)	2.954	1.222	7.143	**0.016**
Radiotherapy		872 (87.6)	1.700	0.777	3.721	0.184
Physical comorbidity		658 (66.1)	0.679	0.454	1.015	0.059
Childhood adversity Exp		44 (4.4)	0.413	0.122	1.393	0.154
Childhood severe illness Exp		51 (5.1)	2.331	1.170	4.643	**0.016**
Family cancer history		254 (25.5)	1.392	0.908	2.133	0.129

*In bold: P < 0.05; Exp, Experience; FCR, Fear of Cancer Recurrence; OR, Odds ratio*.

### Multivariate Logistic Regression Analyses

Multivariate logistic regression analysis indicated that chemotherapy (OR = 2.954, *P* = 0.016), and childhood severe illness experience (OR = 2.331, *P* = 0.016) were positively associated with high FCR, while higher monthly income (more than 10,000 RMB, OR = 0.403, *P* = 0.046) was negative associated with high FCR (Table 1).

### Network Analysis

#### Network Structure

**Figure 1:**
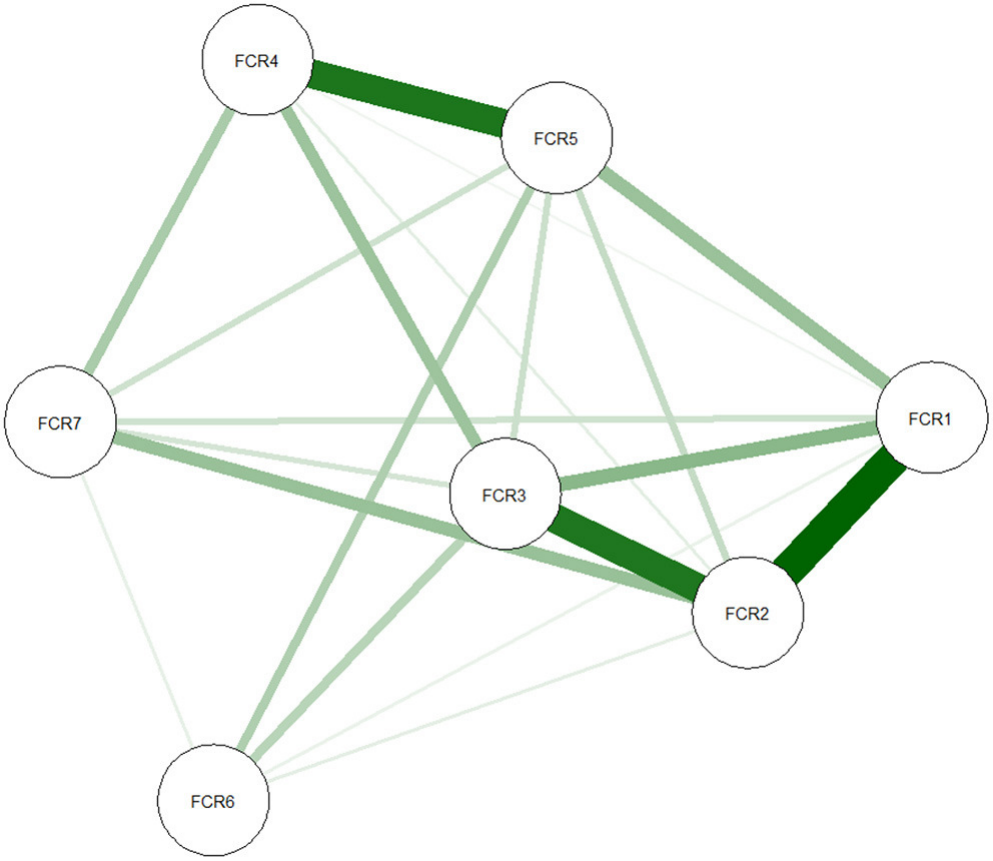
Network of FCR symptoms. In this diagram, nodes with stronger correlations are closer to each other. The thickness of an edge indicates the strength of the correlation. Green lines indicate positive associations. FCR, Fear of Cancer Recurrence.

**Figure 2:**
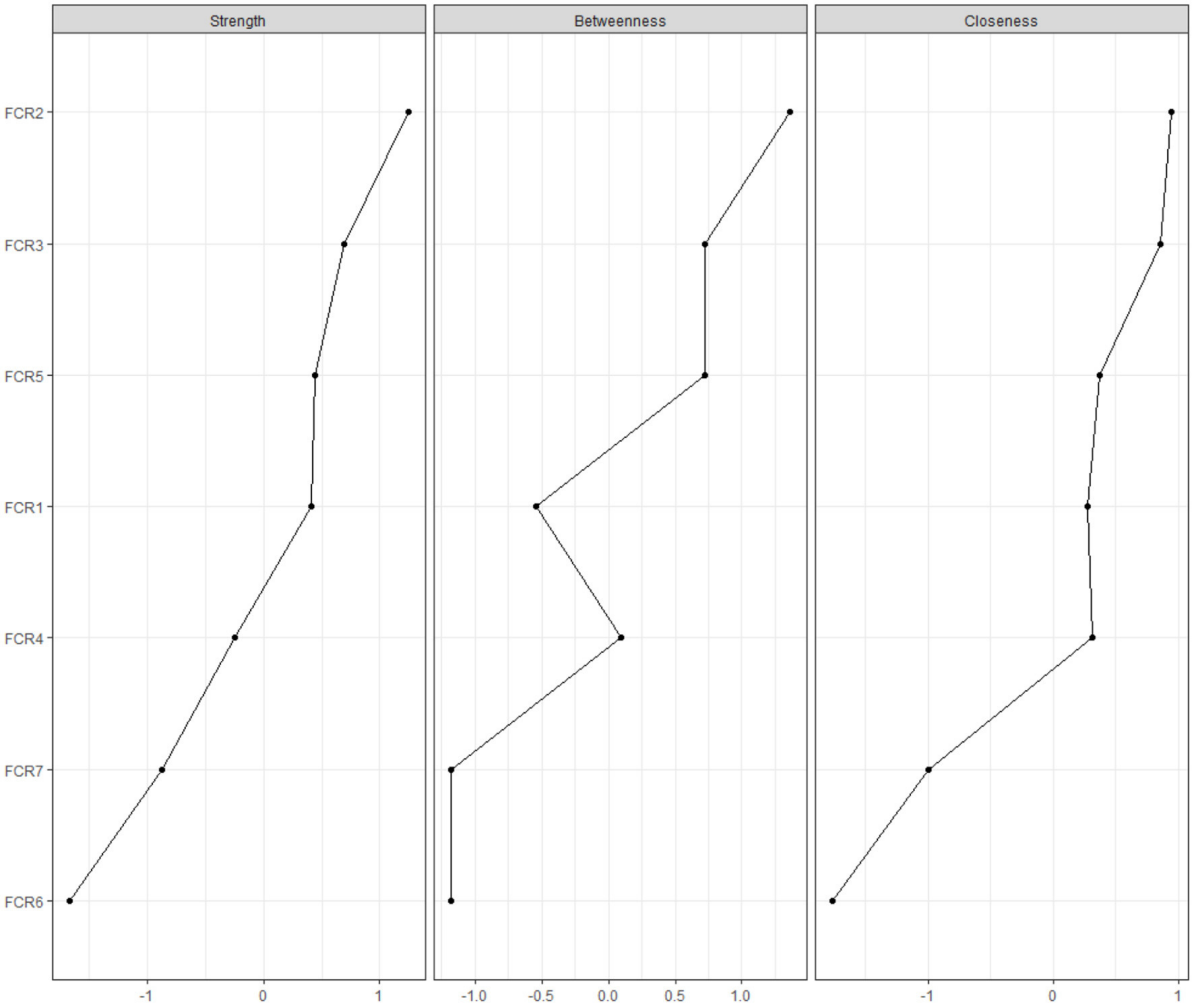
Centrality indices of FCR symptoms. FCR, Fear of Cancer Recurrence.

The cross-sectional network of FCR symptoms, as estimated with the *EBICglasso* model, is shown in Figure 1. The generated network was most well-connected, with no isolated nodes. Out of 21 edges, 20 of them were estimated to be nonzero. The edge between FCR1 and FCR2 (“*Afraid of recurrence”-“Worried/anxious about recurrence”*) was the thickest and most saturated edge in the network. Figure 2 shows the network centrality indices. The node #FCR2 (“*Worried/anxious about recurrence”*, Strength = 1.190, Betweenness = 4, and Closeness = 0.026) was the most central node within the network (with more direct connections with other nodes). Node #FCR6 (“*Examining for physical signs”*) was situated at the periphery of the model and was the least central node in the network (Strength = 0.373, Betweenness = 0, and Closeness = 0.015) (Table 2).

**Table 2:** Centrality of FCR items.

**Items**	**Strength**	**Betweenness**	**Closeness**	**EI**
Q1: I am afraid that my cancer may recur	0.956	1	0.023	0.956
Q2: I am worried or anxious about the possibility of cancer recurrence	1.190	4	0.026	1.190
Q3: How often have you worried about the possibility of getting cancer again	1.034	3	0.026	1.034
Q4: I get waves of strong feelings about the cancer coming back	0.772	2	0.024	0.772
Q5: I think about the cancer returning when I did not mean to	0.965	3	0.024	0.965
Q6: I examine myself to see if I have physical signs of cancer	0.373	0	0.015	0.373
Q7: To what extent does worry about getting cancer again spill over or intrude on your thoughts and activities	0.597	0	0.018	0.597

*EI, Expected Influence; FCR, Fear of Cancer Recurrence*.

#### Network Stability and Accuracy

**Figure 3:**
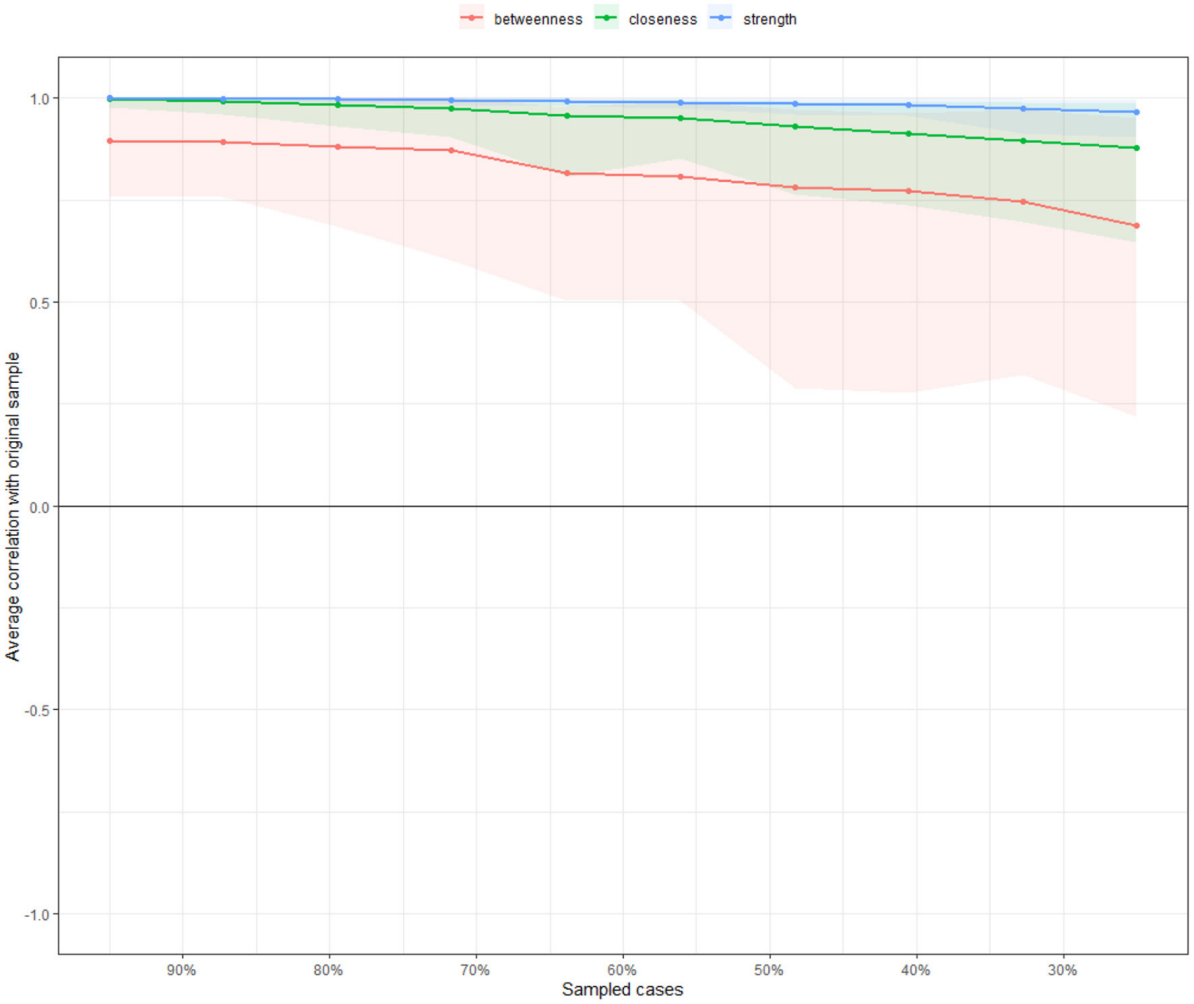
Stability of centrality indices by case dropping subset bootstrap. The x-axis represents the percentage of cases in the original sample used at each step. The y-axis represents the average of correlations between the centrality indices in the original network and the centrality indices in the networks that were re-estimated after dropping increasing percentages of cases. Color areas indicate 95% CI.

Figure 3 shows the results of the case-dropping subset bootstrapping test. The strength and closeness indices remained highly stable (both CS-coefficient = 0.75); however, the betweenness had relatively low stability (CS-coefficient = 0.206). Bootstrapped 95% CIs for the estimated edge-weights suggests that the estimates were reliable and accurate (Supplementary Figure 1). Significance tests of edge weight differences indicated that the edge FCR1-FCR2 (“*Afraid of recurrence”-“Worried/anxious about recurrence”*) was the thickest and most saturated edge in the network and was significantly stronger than most other edges in the model (Supplementary Figure 2) (19). Meanwhile, significance tests of differences in strength indicated that #FCR2 (“*Worried/anxious about recurrence”*) was the most influential node and was significantly stronger than other nodes in the network (Supplementary Figure 3).

#### Network Model Controlling for Age and Gender

After controlling for age and gender, the network model and the local structure indexes were re-estimated. As compared with the original network, an almost identical network was obtained with respect to edges magnitude [*r* = 0.988 (0.970, 0.995)], and strength [*r* = 0.995 (0.970, 0.999)] (Supplementary Figures 4, 5).

## Discussion

This is the first study to investigate the phenomenon of FCR by means of network analysis. First, we found that 54.52% patients reported “moderate level” of FCR, and 13.76% reported “high” FCR. Until now, no similar studies using the 7-item FCR questionnaire were conducted in China, therefore, direct comparisons were difficult to be made. Prevalence of FCR have varied greatly across studies due to different assessment times, measurement instruments, cut-offs, and socio-economic background or clinical status of participants. For example, a study in Hong Kong using the Fear of Cancer Recurrence Inventory-Short Form (FCRI-SF) found that about 26.0% of the participants reported subclinical FCR, and 11.2% of them reported clinically significant FCR (34), while another study in mainland China found that 76.81% of the breast cancer patients experienced clinically significant FCR, using the same assessment tool (35). Based on the findings of the current study, it is suggested that FCR is common among Chinese cancer patients, and most of the patients experienced moderate FCR.

We found that chemotherapy and childhood severe illness experience was positively associated with high FCR. It has been consistently found that patients with chemotherapy (13, 25) or previous adverse experience (36) are at increased risk of experience emotional distress, such as, anxiety, depression or insomnia (37). Therefore, it is possible that these populations are more vulnerable to develop FCR when facing cancer diagnosis and treatment (7). Meanwhile, higher income level was found to be negatively associated with FCR (38). Existing evidence have indicated that economic burden is one of the most stressful live events for an individual, which may lead to depression or other psychological disturbances (39). It is possible that compared to those who are struggling financially (i.e., treatment fees), individuals with better household economic support might feel more confident about their future, and feel less anxious or uncertain. On the contrary, cancer patients with lower monthly income might be more worried that the recurrence of their disease would aggravate the financial burden on their families (38).

To better understand FCR, this study conducted a thorough examination of the inner structure of FCR by network analysis. It has been suggested that higher centrality values are representative of greater importance within the network, and symptoms with high centrality measures may be important symptoms as potential targets for further novel, focused interventions (29). Researchers also suggest that compared to peripheral nodes, central nodes in a network model are more important predictors of clinical outcomes (19). The key finding of the network analysis is that node #FCR2 *(“Worried/anxious about recurrence”*) was the central node in the network, while node #FCR6 (“*Examining for physical signs”*) was the least central node in the model. Our findings are consistent with the key characteristics of clinical FCR, that is, “*persistent and high levels of preoccupation and/or worry*” (6, 40).

FCR is considered as a complex experience involving emotional, perceptual, conceptual, bodily and behavioral dimensions (14). According to previous study, within the 7-item FCR scale, item one to four was designed to feature anxiety, worry, and strong feelings coupled with return of cancer, item five and seven focus on the cognitive processing component that anxiety, fears or worries may interfere or potentially distort thoughts about cancer returning, and item six was used to measure behavioral response to FCR (25). Our findings indicated that compared to behavioral component (i.e., body checking, overscreening and overtreatment) and cognitive component (i.e., intrusions), emotional component (i.e., worry/anxious) features as crucial issue for identification in patients with FCR and might be potential targets for further interventions. These findings are consistent with the definition of FCR as “*fear, worry, or concern relating to the possibility that cancer will come back or progress*” (40), which emphases the importance of an individual's emotional distress. Additionally, these results are partially consistent with a recent meta-analysis of randomized controlled trials, which found larger post-intervention effects for CBT when focusing on the processes of emotion and cognition, such as worry, rumination and attention bias (15).

Our study indicated that after controlling for gender and age, both network edges and the strength centrality metric remained stable, which means the two confounders did not significantly influence the network structure, and it improved the reliability in drawing conclusions from the current network (21).

It is important to mention that one limitation of the study is, data in the current study were cross-sectional in nature. Therefore, no directionality among items could be derived. Longitudinal studies should be carried out to investigate the changes between symptoms over time. Second, the results should be interpreted with caution as the generated networks are based on group-level analysis, and we are uncertain whether group-level results are representative for individuals (41). Third, all findings in the current study were based on self-reported data, and we could not rule out the possibility of recall bias. Fourth, certain factors that may influence an individual's FCR, such as, social support, family relationship, changes in hormones, disease severity, disease prognosis, and substances use etc. was not examined in this study.

Future network studies could focus more on the relationship between FCR and related psychological disturbances, such as depression, anxiety and post-traumatic stress symptoms, to assess the “bridge symptoms/items” that mediate the interaction among different disorders/syndromes. Examining FCR, anxiety, and depression symptoms as dynamic systems may provide new insights into the maintenance of these psychological problems. Further longitudinal studies could also help to better understand the directionality of these bridge pathways. There is a possibility of a bidirectional relationships such that FCR could predict more severe anxiety/depression, and that anxiety/depression could lead to higher FCR. It would also be interesting to investigate the association between FCR items and potential mental and social consequences. If the activation of one FCR symptom/item predicts the activation of a negative outcome, or the deactivation of one FCR symptoms/item leads to the absence of a certain outcome, these items would be prime candidates for future intervention research.

## Conclusion

In conclusion, this study showed that fear of recurrence is a frequently reported issue among Chinese cancer patients. Compared to behavioral component (i.e., body checking, overscreening and overtreatment) and cognitive component (i.e., intrusions), emotional component (i.e., worry/anxious) is more central to identify FCR and might be potential targets for further interventions. Patients with chemotherapy and childhood severe illness experience were more vulnerable and should be particularly monitored. As patients with high FCR could be influenced in their well-being, health-related quality of life, and emotional and social functioning, regular FCR assessment should be adopted. Flexible clinical interventions, such as community reinforcement, and psychotherapy focusing on the processes of emotion and cognition, could be beneficial for patients with high FCR.

## Data Availability Statement

The raw data supporting the conclusions of this article will be made available by the authors, without undue reservation.

## Ethics Statement

The studies involving human participants were reviewed and approved by Southern Medical University Nanfang Hospital (Ref No. NFEC-2018-038). The patients/participants provided their written informed consent to participate in this study.

## Author Contributions

XL, YY, and BZ: study design. WL, YC, HS, TL, and JZ: data collection, analysis and interpretation. XL, WL, YC, and HS: manuscript drafting. GH, YY, and BZ: manuscript revision. All co-authors approved the final version for publication.

## Funding

This research was supported in part by the National Natural Science Foundation of China (Nos. 72101107 and 82071488), Guangzhou Science and Technology Project (Nos. 201904010326 and 201804010132), Clinical Research Program of Nanfang Hospital, Southern Medical University (2021CR014), Construction project of Team Construction in Guangdong Province Teaching Quality and Teaching Reform Project ([2020]19), College Innovation and Entrepreneurship (Employment) Education Project in Guangzhou ([2019]15), and Guangdong Province Degree and Postgraduate Education Reform Research Project (2021JGXM026).

## Conflict of Interest

The authors declare that the research was conducted in the absence of any commercial or financial relationships that could be construed as a potential conflict of interest.

## Publisher's Note

All claims expressed in this article are solely those of the authors and do not necessarily represent those of their affiliated organizations, or those of the publisher, the editors and the reviewers. Any product that may be evaluated in this article, or claim that may be made by its manufacturer, is not guaranteed or endorsed by the publisher.
