# Supplementary material for: Fear of Recurrence in Chinese Cancer Patients: Prevalence, Correlates, and Network Analysis
Source: Front Psychiatry. 2022 Feb 7;13:803543. doi: 10.3389/fpsyt.2022.803543 (PMC8859333; doi:10.3389/fpsyt.2022.803543)
Supplement: Supplementary file 1 [file Data_Sheet_1.docx]

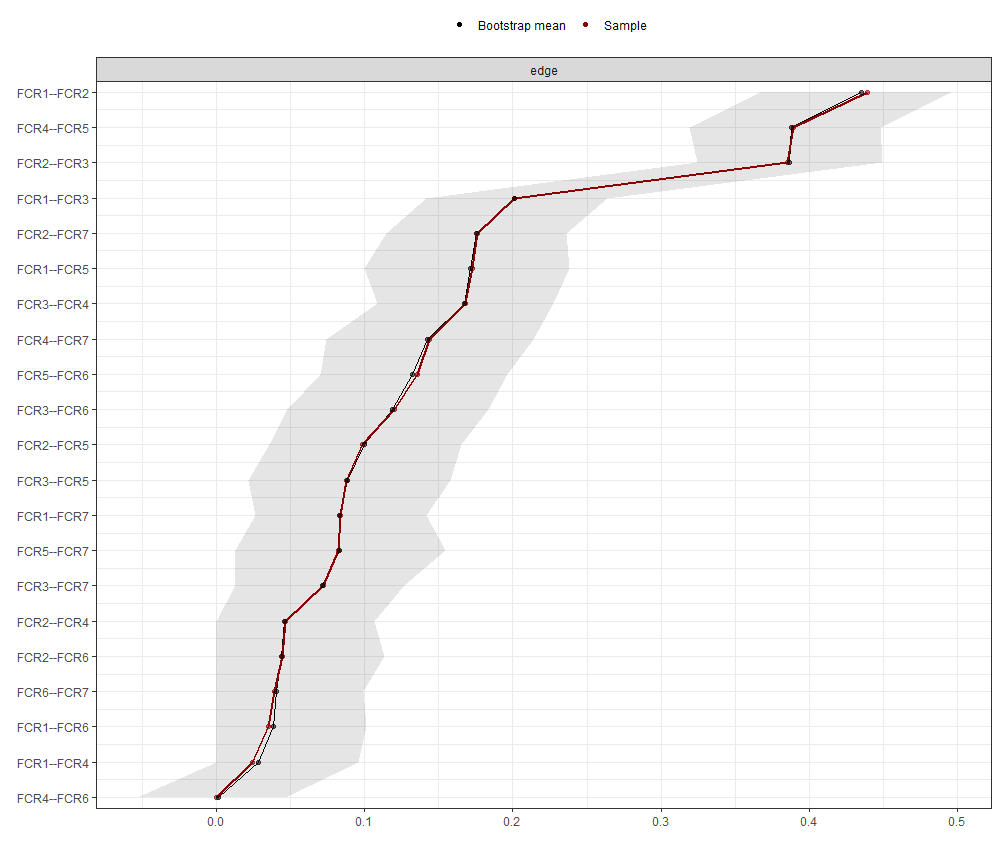


**Supplementary Figure 1 Bootstrapped 95%CIs of estimated edge weights**

Note: The red dots indicate the values of each edge weight, ordered from the highest to the lowest edge-weight values. The gray area represents the 95% CIs of edge weights, estimated with the non-parametric bootstrap procedure. Wide intervals indicate lower stability and narrow intervals indicate higher stability.


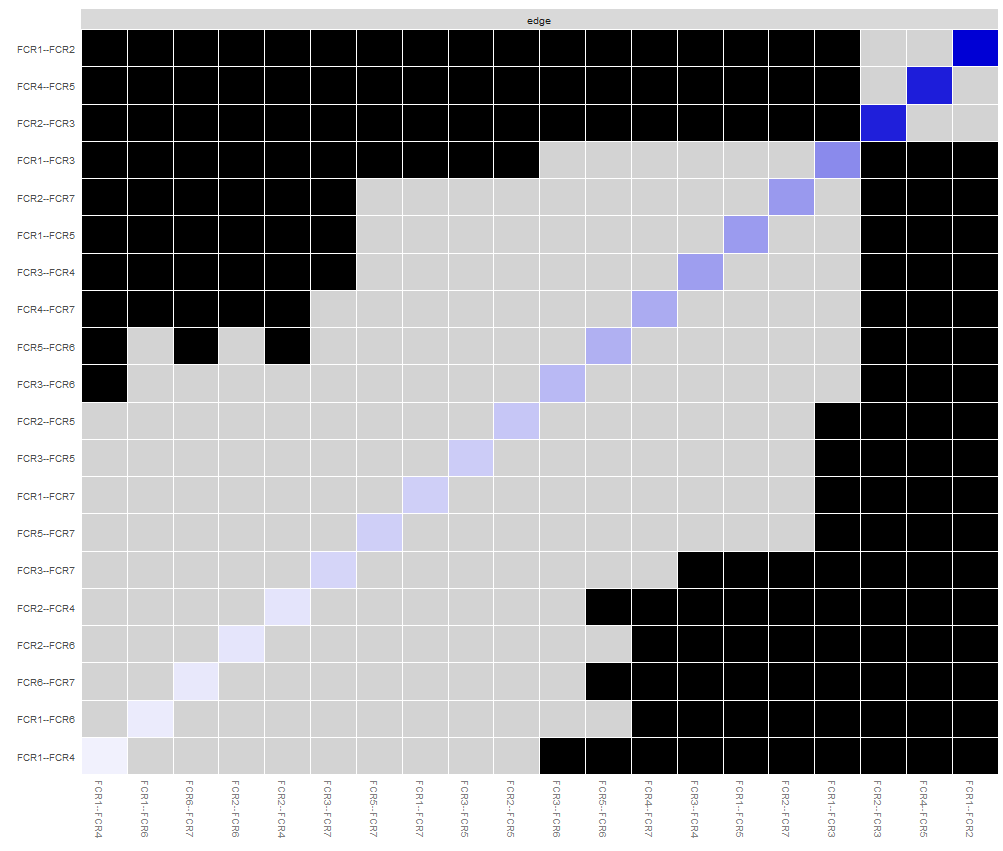


**Supplementary Figure 2 Estimation of edge weight difference by bootstrapped difference test**

Note: Gray boxes represent the edges do not significantly differ from one-another, and black boxes represent edges that do differ significantly from one-another. The diagonal line indicates the strength of edge-weights, shifting from red (representing negative associations) to dark blue (representing positive associations).


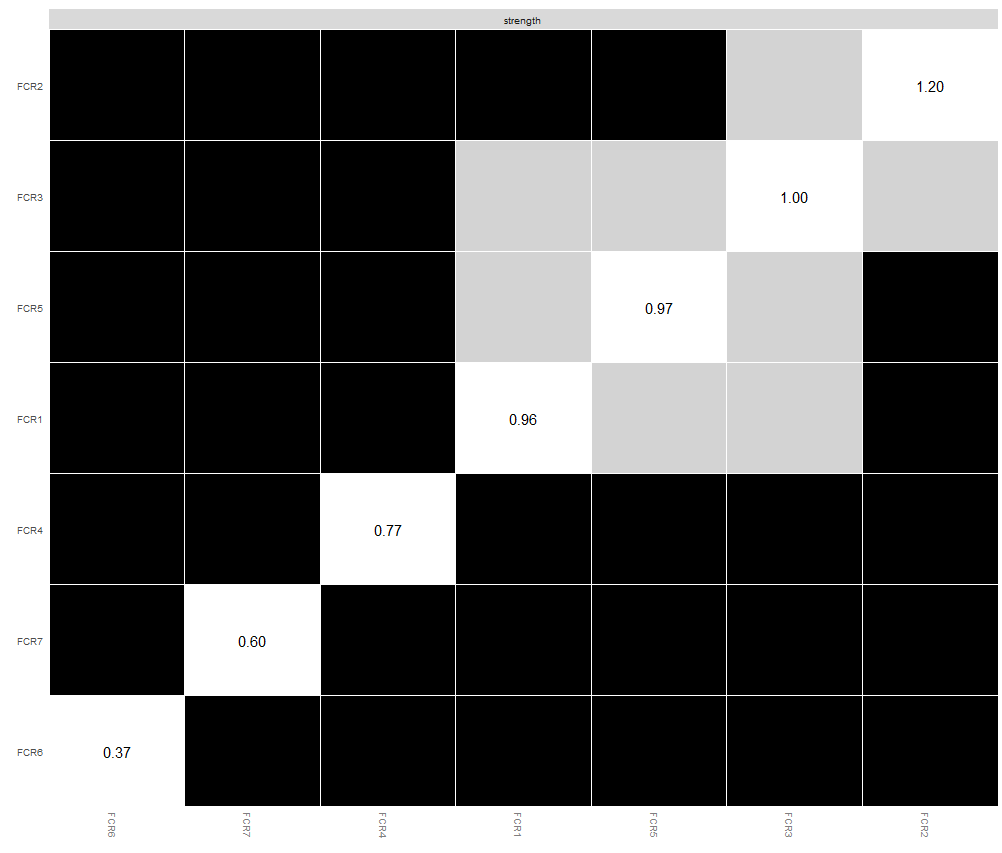


**Supplementary Figure 3. Nonparametric bootstrapped difference test for strength**

Note: Gray boxes indicate no difference between nodes, whereas black boxes indicate significant difference (α = 0.05). Values reported in the diagonal represent the strength values of each node.


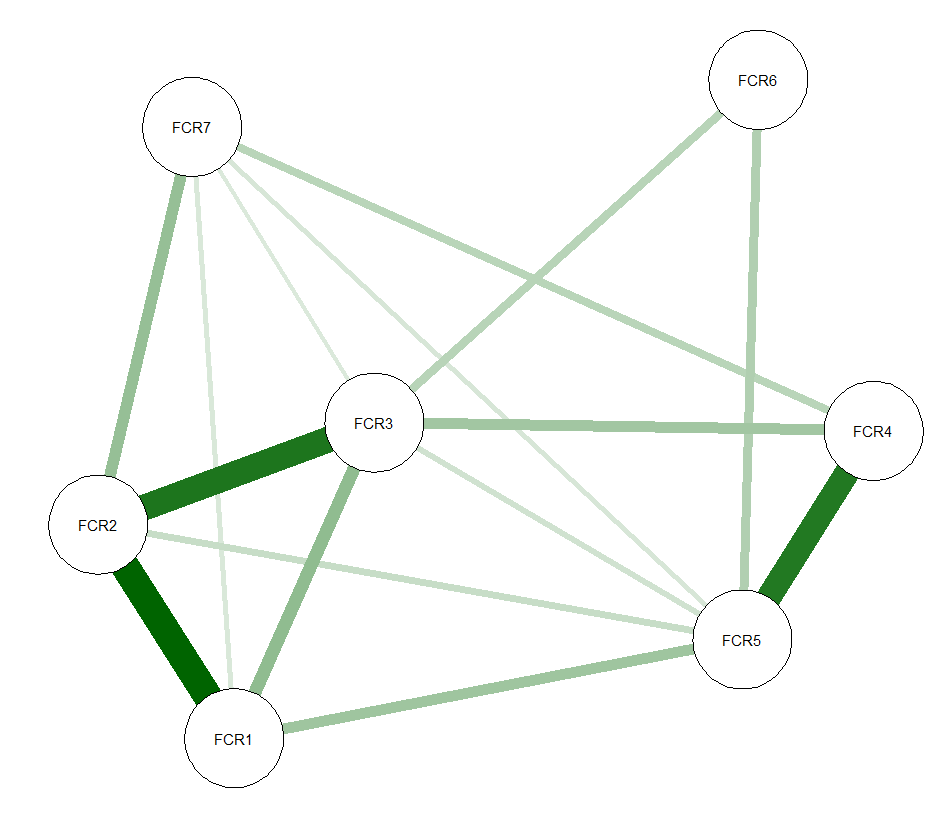


**Supplementary Figure 4 Network of FCR symptoms controlling for age and gender**

Note: In this diagram, nodes with stronger correlations are closer to each other. The thickness of an edge indicates the strength of the correlation. Green lines indicate positive associations. FCR: Fear of Cancer Recurrence.


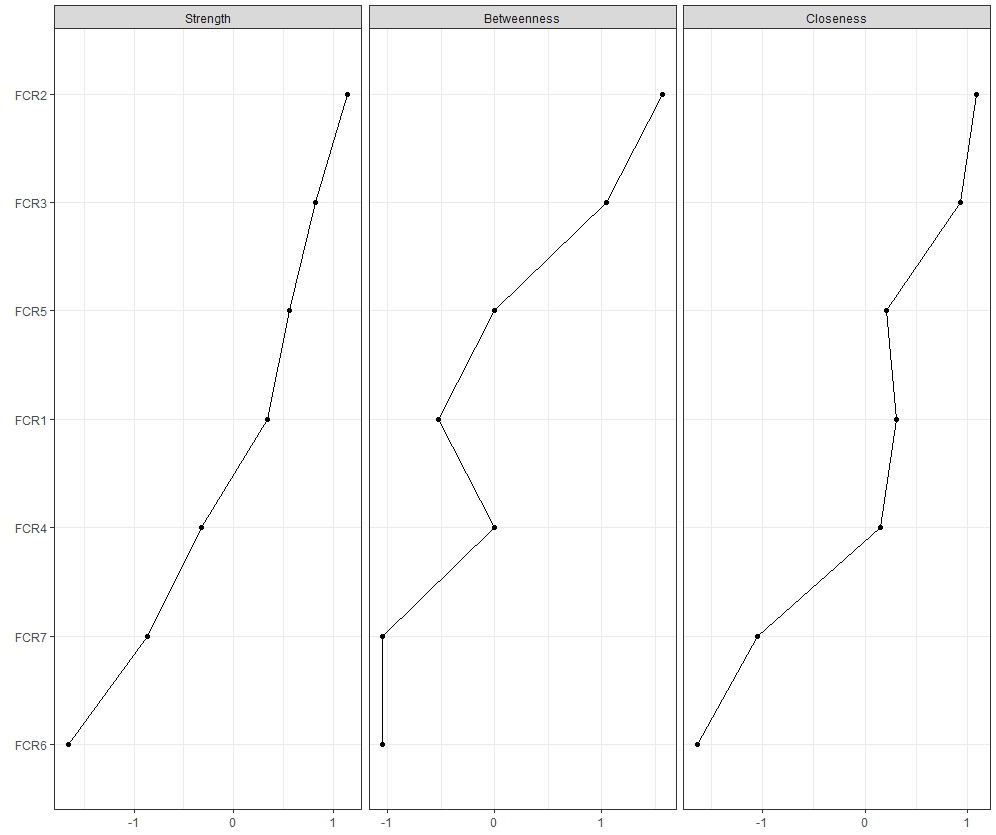


**Supplementary Figure 5 Centrality indices of FCR symptoms controlling for age and gender**

Note: FCR: Fear of Cancer Recurrence.

**Supplementary table 1. Means and standard deviations of FCR items**

| **Item** | **N** | **Minimum** | **Maximum** | **Mean** | **SD** |
| --- | --- | --- | --- | --- | --- |
| FCRQ1 | 996 | 1 | 5 | 2.955 | 1.052 |
| FCRQ2 | 996 | 1 | 5 | 2.775 | 1.001 |
| FCRQ3 | 996 | 1 | 5 | 2.689 | 0.879 |
| FCRQ4 | 996 | 1 | 5 | 2.019 | 0.955 |
| FCRQ5 | 996 | 1 | 5 | 2.421 | 0.897 |
| FCRQ6 | 996 | 1 | 5 | 2.928 | 0.943 |
| FCRQ7 | 996 | 1 | 10 | 4.143 | 2.225 |
| Overall | 996 | 7 | 40 | 19.928 | 6.347 |
| Note: FCR: Fear of Cancer Recurrence; SD: standard deviation | | | | | |
